# C-reactive protein to serum calcium ratio as a novel biomarker for predicting severity in acute pancreatitis: a retrospective cross-sectional study

**DOI:** 10.3389/fmed.2025.1506543

**Published:** 2025-02-07

**Authors:** Xinqi Chen, Yisen Huang, Qiaoli Xu, Bifeng Zhang, Yubin Wang, Meixue Huang

**Affiliations:** Department of Gastroenterology, First Hospital of Quanzhou Affiliated to Fujian Medical University, Quanzhou, Fujian, China

**Keywords:** acute pancreatitis, C-reactive protein to serum calcium ratio, severity, biomarker, cross-sectional study

## Abstract

**Background:**

Acute pancreatitis (AP) is a prevalent gastrointestinal emergency with a wide spectrum of clinical outcomes, varying from mild cases to severe forms. The early identification of high-risk patients is essential for improving prognosis. However, the predictive and prognostic potential of the C-reactive protein to serum calcium ratio (CCR) in AP has not been investigated. This study aims to explore the association between CCR and disease severity in patients with AP.

**Methods:**

This retrospective cross-sectional study included 476 AP patients. The CCR was calculated from C-reactive protein and serum calcium levels within the first 24 h of admission. Multivariable logistic regression models were used to evaluate the relationship between CCR and AP severity, with restricted cubic spline analysis and receiver operating characteristic (ROC) analysis to assess dose–response and predictive performance, respectively.

**Results:**

Of the 476 patients, 176 (37%) had mild acute pancreatitis (MAP) and 300 (63%) had moderate to severe AP. The CCR distribution had a median value of 17.5, with an interquartile range (IQR) of 3.0 to 60.2. Each unit increase in CCR was associated with a 7% increase in the risk of developing moderate to severe AP (OR: 1.07; 95% CI: 1.06–1.09). In fully adjusted models, this association remained statistically significant. The area under the curve (AUC) for CCR in predicting moderate to severe AP was 86.9%, with a sensitivity of 73.7% and specificity of 89.2%.

**Conclusion:**

The CCR measured within the first 24 h of admission shows promise as a valuable biomarker for predicting the severity of AP. However, further multicenter prospective cohort studies are needed to confirm its clinical utility and investigate its role in improving treatment strategies and patient management.

## 1 Introduction

Acute pancreatitis (AP) is a prevalent gastrointestinal emergency, with an annual incidence ranging from 13 to 45 cases per 100,000 individuals, marked by its rapid onset and potential for severe complications (1). The primary causes of AP comprise biliary tract disease, excessive alcohol consumption, and hypertriglyceridemia (2–4). The pathophysiology of AP involves the premature activation of pancreatic enzymes, leading to autodigestion of pancreatic tissue and triggering an inflammatory cascade that extends beyond the pancreas to affect distant organs (5). While the majority of AP cases resolve with supportive care, approximately 20% of patients progress to moderate to severe AP, characterized by persistent organ failure and local complications such as necrosis or abscess formation (6–8). Early identification of high-risk patients and appropriate adjustment of therapeutic strategies are crucial for improving patient outcomes. Consequently, the discovery of simple, accurate indicators to predict AP severity is of significant clinical value.

Since the 1970s, multifactorial scoring systems, such as the AP-specific Ranson score (9), Bedside Index of Acute Pancreatitis Severity (BISAP) (10), computed tomography severity index (CTSI) (11), and the broader Acute Physiology and Chronic Health Examination (APACHE)-II (12), have been widely employed to assess the severity of acute pancreatitis (AP). These scoring systems have become critical tools in evaluating AP severity and guiding clinical decision-making. However, systems with a greater number of indicators can be challenging to apply in real-time due to their complexity and the need for multiple parameters. As a result, there is growing interest in simpler laboratory markers that can effectively evaluate AP severity, predict complications, and assess potential mortality risks.

Recently, novel combined indicators, such as the lactate to albumin Ratio (LAR) (1), C-reactive protein to lymphocyte ratio (CLR) (13), and neutrophil to lymphocyte ratio (NLR) (14), have been utilized for prognostic evaluation in patients with AP. Despite these advances, research on the predictive and prognostic potential of the C-reactive protein to serum calcium ratio (CCR) in AP remains limited. This study aims to explore the association between CCR and disease severity in AP patients, addressing a critical gap in current research.

## 2 Methods

### 2.1 Study population

This retrospective cross-sectional study involved in-patients with AP who were admitted to the First Hospital of Quanzhou Affiliated to Fujian Medical University between January 2018 and December 2019. All enrolled patients fulfilled the diagnostic criteria set by the Atlanta classification (15). Mild acute pancreatitis (MAP) is defined by the lack of organ failure and absence of local or systemic complications. Moderately severe acute pancreatitis (MSAP) involves transient organ failure or local/systemic complications without persistent organ failure. Severe acute pancreatitis (SAP) is marked by persistent organ failure. The exclusion criteria were as follows: (1) chronic pancreatitis, (2) malignant tumors, (3) pregnancy, and (4) incomplete data. In total, 476 patients with AP were included in the analysis, comprising 176 with MAP and 300 with moderate to severe AP. This study adhered to the Declaration of Helsinki and received approval from our institution’s Ethics Committee (QYL 2021–199). Given its retrospective nature and use of anonymized data, informed consent was waived.

### 2.2 Data collection

All patient demographic and laboratory information was extracted from our hospital’s electronic medical records. The collected data encompassed age, gender, smoking and alcohol consumption, preexisting conditions, disease severity, length of hospital stay, and laboratory results from the first 24 h after admission.

### 2.3 Measurement of CCR

The CCR was determined using the formula: CCR (mg/mmol) = C-reactive protein (CRP) level (mg/L)/serum calcium (mmol/L).

### 2.4 Statistical analysis

Continuous variables were reported as the mean and standard deviation (SD) for data following a normal distribution, or as the median and interquartile range (IQR) for non-normally distributed data. Categorical variables were reported as percentages (%). Differences between groups were analyzed using one-way ANOVA for normally distributed continuous variables, and chi-square or trend tests for categorical variables.

Multivariate logistic regression models were employed to evaluate the odds ratios (OR) and 95% confidence intervals (CI) for the relationship between CCR and the incidence of moderate to severe AP. Variables included in the model were chosen based on their clinical relevance, statistical significance in univariable analyses, and a change in the estimated effect of at least 10% that could influence potential confounding effects (16). The regression analysis involved four models. Model 1 had no adjustments, while Model 2 was adjusted for age and gender. In Model 3, additional adjustments were made for smoking status, alcohol use, diabetes, fatty liver, etiology, and length of hospital stay. Model 4 further adjusted for hemoglobin and albumin levels on top of the factors in Model 3.

Restricted cubic spline analyses were used to explore the dose–response relationship between CCR and the incidence of moderate to severe AP.

Subgroup analyses were performed by stratifying according to relevant covariates, including age, gender, smoking status, alcohol use, diabetes, fatty liver disease, and etiology.

Receiver Operating Characteristic (ROC) analysis was employed to evaluate the predictive performance, sensitivity, and specificity of CCR for moderate to severe AP incidence. The optimal threshold for CCR was established using the Youden Index.

All analyses were conducted with the statistical software R 3.3.2 (http://www.R-project.org, The R Foundation) and Free Statistics software version 1.9. A *p*-value of less than 0.05 (two-sided) was considered statistically significant.

## 3 Results

### 3.1 Baseline characteristics

**Figure 1:**
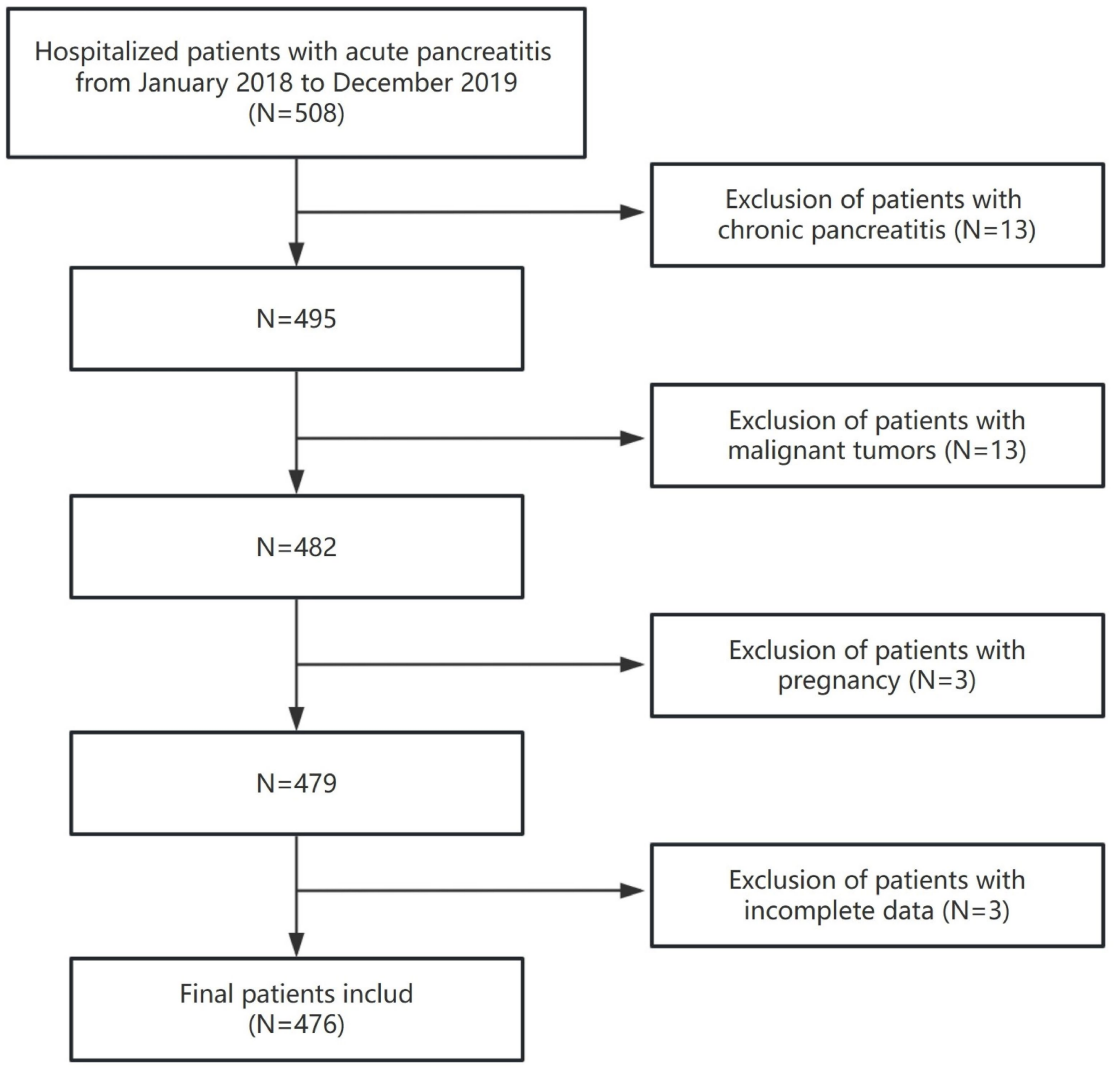
The flow chart of patient selection.

The study population selection is illustrated in Figure 1, with a total of 476 patients being included. The baseline characteristics of the participants, categorized by CCR tertiles, are presented in Table 1. The enrolled patients were stratified into three tertiles based on their CCR levels: Q1 with 2.0 (1.0, 3.0) mg/mmol, Q2 with 17.5 (9.9, 30.0) mg/mmol, and Q3 with 82.8 (60.4, 99.3) mg/mmol. Among the 476 patients, the average age was 44 ± 13.2 years, with 364 (76.5%) of the participants being male. The CCR distribution had a median value of 17.5, with an IQR of 3.0 to 60.2. The CCR levels were higher in men compared to women (133 [83.6%] vs. 115 [72.3%]), in smokers compared to non-smokers (67 [42.1%] vs. 49 [30.8%]), and in drinkers compared to non-drinkers (81 [50.9%] vs. 61 [38.4%]). Additionally, a higher CCR was associated with prolonged hospitalizations, increased rates of diabetes and fatty liver disease, as well as a higher incidence of moderate to severe AP.

**Table 1:** Baseline characteristics of the patients with acute pancreatitis.

Covariates	Total (*n* = 476)	Q1 (*n* = 159)	Q2 (*n* = 158)	Q3 (*n* = 159)	*p* value
CCR, mg/mmol	17.5 (3.0, 60.2)	2.0 (1.0, 3.0)	17.5 (9.9, 30.0)	82.8 (60.4, 99.3)	< 0.001
Age, years	44.0 ± 13.2	45.3 ± 13.1	45.1 ± 14.0	41.7 ± 12.2	0.026
Gender					0.032
Male	364 (76.5)	115 (72.3)	116 (73.4)	133 (83.6)	
Female	112 (23.5)	44 (27.7)	42 (26.6)	26 (16.4)	
Smoking	161 (33.8)	49 (30.8)	45 (28.5)	67 (42.1)	0.023
Alcohol consumption	196 (41.2)	61 (38.4)	54 (34.2)	81 (50.9)	0.007
Diabetes	154 (32.4)	31 (19.5)	57 (36.1)	66 (41.5)	< 0.001
Fatty live	280 (58.8)	67 (42.1)	101 (63.9)	112 (70.4)	< 0.001
Etiology					< 0.001
Hypertriglyceridemia	277 (58.2)	68 (42.8)	94 (59.5)	115 (72.3)	
Biliary	92 (19.3)	36 (22.6)	34 (21.5)	22 (13.8)	
Alcohol	53 (11.1)	26 (16.4)	14 (8.9)	13 (8.2)	
Other	54 (11.3)	29 (18.2)	16 (10.1)	9 (5.7)	
Hospitalization days, days	6.0 (5.0, 9.0)	5.0 (4.0, 7.0)	6.0 (5.0, 8.0)	8.0 (6.0, 11.0)	< 0.001
Moderate to severe AP	300 (63.0)	40 (25.2)	105 (66.5)	155 (97.5)	< 0.001
WBC, 10^9^/L	11.9 (9.3, 15.4)	10.1 (7.6, 12.0)	11.9 (9.8, 15.4)	14.3 (11.6, 17.1)	< 0.001
Hemoglobin, g/L	151.0 ± 22.9	146.9 ± 22.0	152.5 ± 23.8	153.8 ± 22.4	0.017
Platelets, 10^9^/L	220.0 (181.0, 261.0)	225.0 (182.0, 268.0)	218.5 (171.2, 257.8)	220.0 (186.5, 253.5)	0.576
Serum amylase, U/L	246.5 (120.8, 627.5)	286.0 (132.0, 696.0)	234.0 (109.0, 676.0)	245.0 (117.5, 528.5)	0.189
Albumin, g/L	38.8 ± 16.9	41.8 ± 28.0	38.5 ± 5.2	36.0 ± 5.6	0.008
AST, U/L	27.0 (19.8, 44.0)	28.0 (20.0, 40.0)	27.5 (21.0, 47.0)	26.0 (19.0, 47.0)	0.459
ALT, U/L	27.0 (18.0, 45.2)	27.0 (18.5, 47.5)	30.0 (19.0, 54.0)	27.0 (17.0, 38.5)	0.287
Creatinine, umol/L	65.0 (55.0, 78.0)	64.4 (55.0, 76.0)	67.0 (56.2, 79.0)	64.0 (53.8, 79.0)	0.615
Sodium, mmol/L	133.6 ± 12.9	135.6 ± 11.3	133.4 ± 11.3	131.9 ± 15.5	0.041
Calcium, mmol/L	2.2 (2.1, 2.3)	2.3 (2.2, 2.3)	2.2 (2.2, 2.3)	2.1 (2.0, 2.3)	< 0.001
CRP, mg/L	38.2 (7.0, 126.0)	5.0 (2.4, 7.0)	38.2 (22.0, 67.0)	183.0 (126.0, 200.0)	< 0.001

CCR, C-reactive protein/serum calcium ratio; AP, acute pancreatitis; WBC, white blood cell; ALT, alanine transaminase; AST, aspartate transaminase; CRP, C-reactive protein.

### 3.2 Relationship between CCR and the incidence of moderate to severe AP

Table 2 displays the findings from a multivariable logistic regression analysis, which assessed the association between CCR and the incidence of moderate to severe AP. When CCR was analyzed as a continuous variable, each one-unit rise (1 mg/mmol) was associated with a 7% increase in the risk of developing moderate to severe AP (OR: 1.07; 95% CI: 1.06–1.09). This relationship remained statistically significant after adjusting for covariates in models 2 and 3. In the fully adjusted model (Model 4), which included all covariates, each unit increase in CCR was still linked to a 7% higher risk of developing moderate to severe AP (OR: 1.07; 95% CI: 1.05–1.09).

**Table 2:** Association between C-reactive protein/serum calcium ratio and the incidence of moderate to severe acute pancreatitis.

Exposure	Model 1		Model 2		Model 3		Model 4	
	OR (95% CI)	*P* value	OR (95% CI)	*P* value	OR (95% CI)	*P* value	OR (95% CI)	*P* value
CCR	1.07 (1.06 ~ 1.09)	<0.001	1.07 (1.06 ~ 1.09)	<0.001	1.07 (1.05 ~ 1.09)	<0.001	1.07 (1.05 ~ 1.09)	<0.001
CCR tertiles								
Q1(1.0, 3.0)	Reference		Reference		Reference		Reference	
Q2(9.9, 30.0)	5.89 (3.62 ~ 9.59)	<0.001	6.36(3.84 ~ 10.53)	<0.001	5.10 (2.91 ~ 8.94)	<0.001	4.78 (2.70 ~ 8.45)	<0.001
Q3(60.4, 99.3)	115.28(40.13 ~ 331.16)	<0.001	115.53(39.76 ~ 335.66)	<0.001	78.7(25.57 ~ 242.24)	<0.001	74.95 (24.14 ~ 232.73)	<0.001
P for trend		<0.001		<0.001		<0.001		<0.001

Model 1: no covariates were adjusted. Model 2: adjusted for age and gender. Model 3: adjusted for age, gender, smoking, alcohol consumption, diabetes, fatty liver, etiology and hospitalization days. Model 4: adjusted for age, gender, smoking, alcohol consumption, diabetes, fatty liver, etiology, hospitalization days, hemoglobin and albumin. CCR, C-reactive protein/serum calcium ratio; OR, odds ratios; CI, confidence intervals.

Additionally, in the fully adjusted model (Model 4), when CCR was treated as a categorical variable, a clear trend was observed. Compared to the lowest CCR group (Q1), the adjusted OR for the second (Q2) and third quartiles (Q3) were 4.78 (95% CI: 2.70–8.45) and 74.95 (95% CI: 24.14–232.73), respectively. The trend analysis showed a statistically significant result (*p* < 0.001).

### 3.3 Dose–response relationships

**Figure 2:**
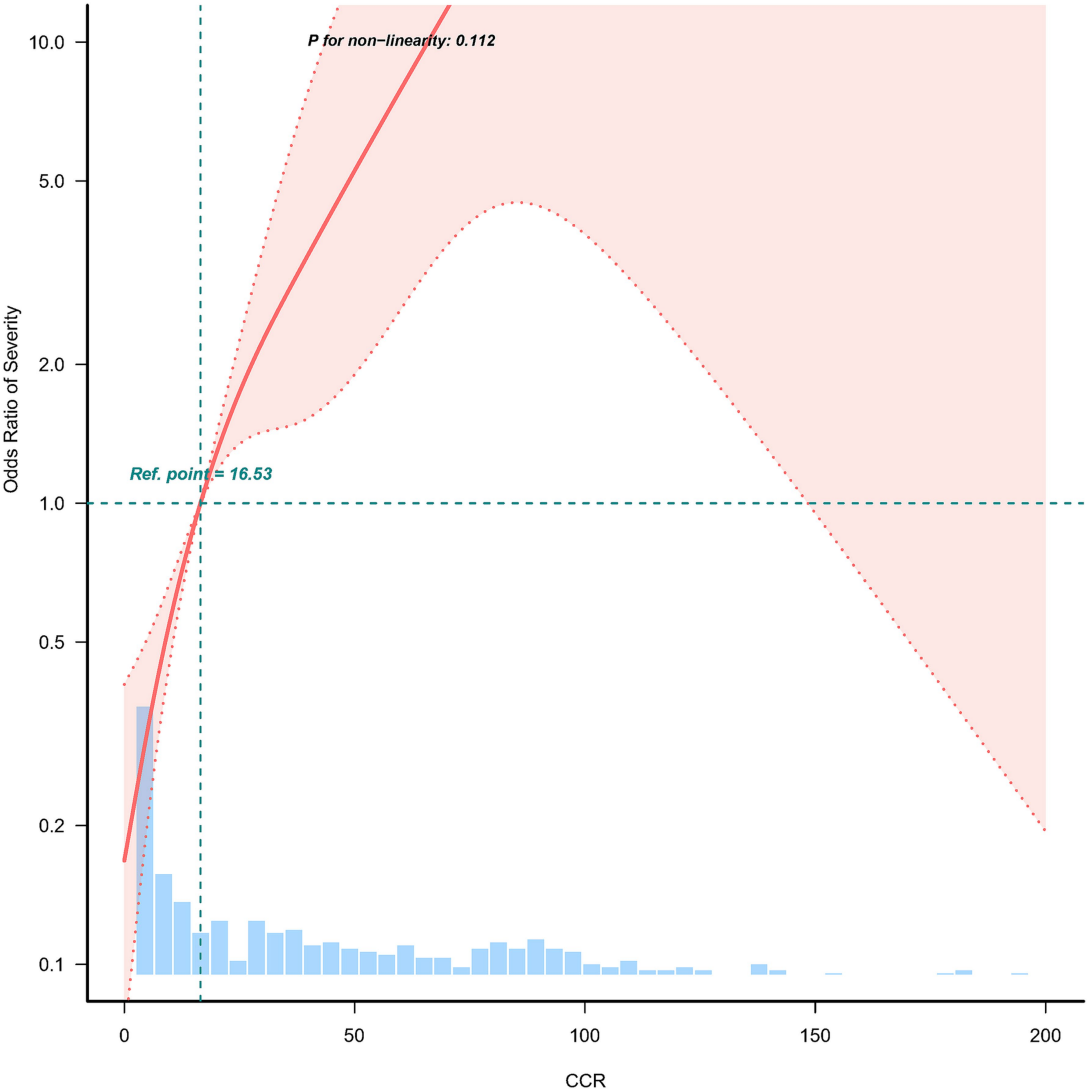
Nonlinear relationship between C-reactive protein/serum calcium ratio and the incidence of moderate to severe acute pancreatitis. Adjusted for age, gender, smoking, alcohol consumption, diabetes, fatty liver, etiology, hospitalization days, hemoglobin and albumin. The red line and the area between the red dashed lines represents the estimated values and their corresponding 95% confidence intervals, respectively.

The study applied a logistic regression model with a cubic spline function to investigate how CCR relates to the incidence of moderate to severe AP. Figure 2 presents the distributions of variables (depicted as blue histograms), the relationship between CCR and the incidence of moderate to severe AP (shown by the solid red line), and the 95% confidence interval (marked by red dashed lines). After adjusting for confounding factors, a significant linear correlation between CCR and moderate to severe AP was found.

### 3.4 Subgroup analyses

**Figure 3:**
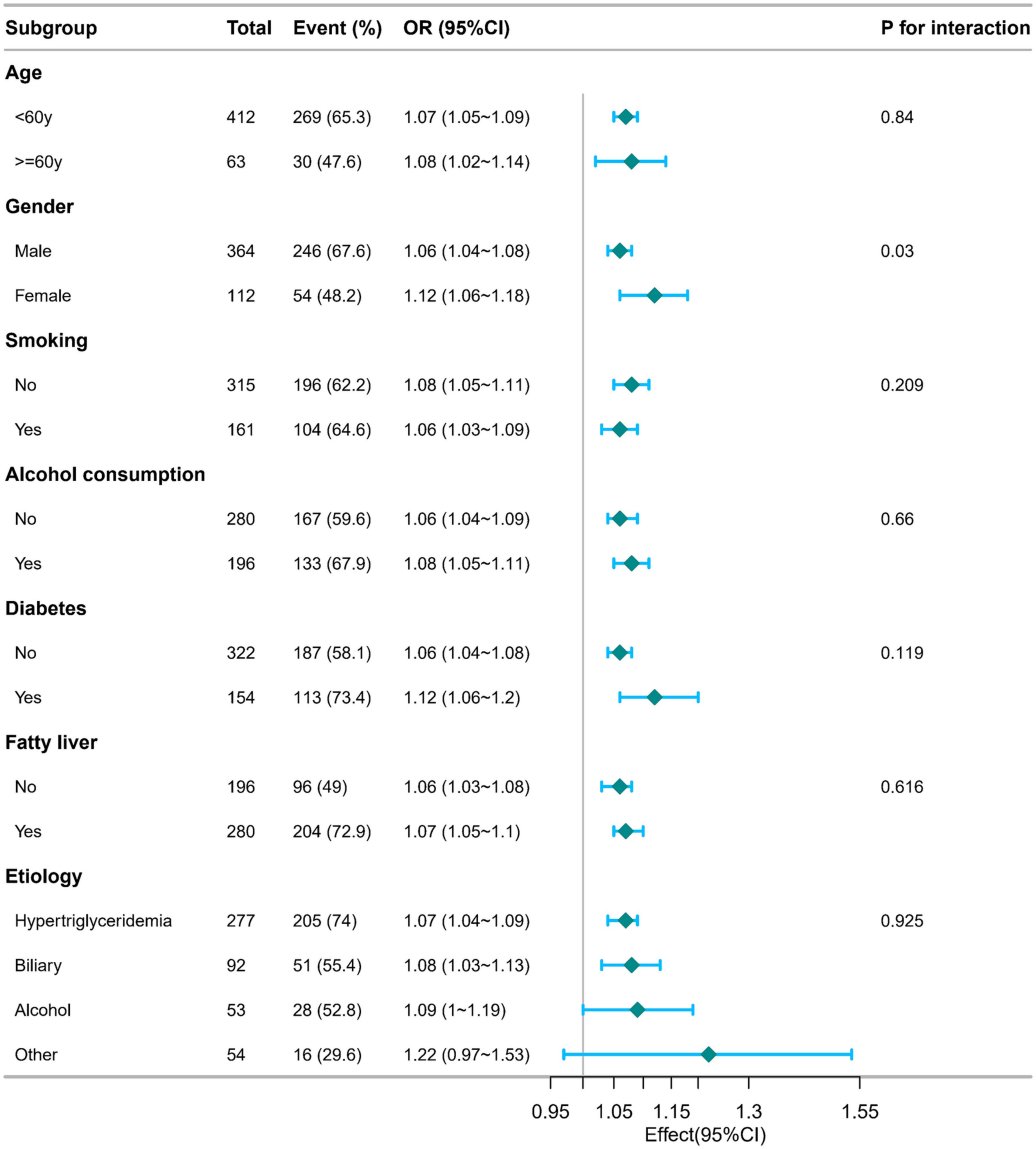
Subgroup analysis of the relationship between C-reactive protein/serum calcium ratio and the incidence of moderate to severe acute pancreatitis. Adjusted for age, gender, smoking, alcohol consumption, diabetes, fatty liver, etiology, hospitalization days, hemoglobin and albumin. OR, odds ratios; CI, confidence intervals.

The study further assessed possible modifiers that might influence the relationship between CCR and the incidence of moderate to severe AP. Stratification was performed based on variables like age, gender, smoking, alcohol consumption, diabetes, fatty liver, and etiology (Figure 3). Nonetheless, no significant interactions were detected.

### 3.5 ROC curve analysis

**Figure 4:**
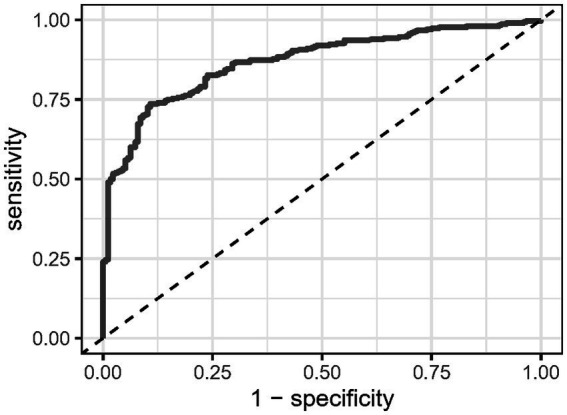
Receiver operating characteristic analysis of C-reactive protein/serum calcium ratio in predicting the onset of moderate to severe acute pancreatitis.

ROC curves were created to evaluate CCR’s predictive accuracy for moderate to severe AP, with detailed results provided in Table 3 and illustrated in Figure 4. The CCR had an area under the curve (AUC) of 86.933% (95% CI: 83.765% ~ 90.100%), and the optimal cut-off value was found to be 16.733. This cut-off achieved a sensitivity of 73.7% and a specificity of 89.2%.

**Table 3:** Information of receiver operating characteristic curve in Figure 4.

Variables	AUC	95%CI	Threshold	Sensitivity	Specificity
CCR	86.933%	83.765% ~ 90.100%	16.733	0.737	0.892

CCR, C-reactive protein/serum calcium ratio; AUC, area under the curve; CI, confidence intervals.

## 4 Discussion

This study demonstrated that the CCR is significantly associated with the severity of AP. Our results show that higher CCR levels are strongly correlated with an increased risk of moderate to severe AP. Specifically, each one-unit increase in CCR was associated with a 7% higher likelihood of developing moderate to severe AP, even after adjusting for relevant confounding factors. Additionally, when patients were stratified into tertiles based on their CCR levels, those in the highest tertile demonstrated a markedly higher risk of moderate to severe AP compared to those in the lowest tertile. These findings suggest that CCR could be a valuable biomarker in predicting AP severity, providing clinicians with a tool for early risk stratification in AP patients. Furthermore, the dose–response relationship between CCR and AP severity, as demonstrated by the restricted cubic spline analysis, underscores the potential clinical utility of CCR in managing this condition.

AP is an inflammatory condition with diverse clinical presentations, varying progression, and different patient outcomes. While mild cases often resolve rapidly, severe cases can induce both local and systemic inflammatory responses, increasing the risk of organ dysfunction or failure, which can be life-threatening. Therefore, promptly identifying high-risk patients who could benefit from intensive care and close monitoring is crucial for effective management. In recent years, researchers have focused on developing systemic inflammatory biomarkers to predict the prognosis of AP patients at an early stage. Prominent biomarkers include the red blood cell distribution width to serum calcium ratio (RDW/Ca) (17), glycemic to lymphocyte ratio (GLR) (18), LAR (1), CLR (13), and NLR (14, 19). These biomarkers are being explored for their potential to offer early insights into disease progression and outcomes. However, no studies have yet explored the association between CCR and AP severity. Thus, this study is the first to evaluate the clinical relevance of CCR in AP.

The association between CRP and AP severity is well-documented, with higher CRP levels consistently linked to worse outcomes, such as necrotizing pancreatitis, organ failure, and increased mortality (20–22). However, CRP alone may not fully capture the metabolic alterations associated with severe AP, particularly hypocalcemia, which is a known marker of poor prognosis in this condition. Previous studies have shown that low serum calcium levels are correlated with increased mortality and disease severity in AP (20, 23, 24). Combining CRP and calcium into a single ratio (CCR) adds a metabolic dimension to the inflammatory marker, providing a more comprehensive assessment of disease severity.

Biologically, the interplay between inflammation and calcium metabolism in AP provides a plausible mechanism for the observed predictive power of CCR. CRP is produced in response to pro-inflammatory cytokines, such as interleukin-6 (IL-6), which are elevated in AP (25). The inflammatory response in severe AP is further exacerbated by the release of pancreatic enzymes that cause local tissue damage and fat necrosis, leading to the consumption of calcium for the formation of calcium soaps (26). This dual process of inflammation and calcium consumption likely drives the progression to more severe disease. The CCR, by combining CRP and serum calcium, effectively reflects both of these pathological processes, making it a robust marker of disease severity. We further evaluated the predictive power of CCR within the first 24 h for determining moderate to severe AP, finding an AUC of 86.933%, indicating strong discriminative ability. Given the simplicity and widespread availability of both CRP and serum calcium measurements in clinical practice, CCR could offer a more practical alternative to complex scoring systems.

The strengths of this study lie in the use of CCR, a novel composite biomarker that evaluates AP severity by integrating inflammation and metabolic disturbances. Furthermore, the large sample size and rigorous multivariable adjustments used in our study enhance the robustness and reliability of our findings. However, there are several limitations to our study. First, the cross-sectional design of the study precludes the establishment of a causal relationship between CCR and the development of moderate to severe AP. Second, as this is a single-center study conducted solely within a Chinese population, the generalizability of our findings to other populations may be limited. Lastly, although we adjusted for several key confounding factors, unmeasured variables may still have influenced the outcomes.

## 5 Conclusion

The CCR measured within the first 24 h of admission shows promise as a valuable biomarker for predicting the severity of AP. However, further multicenter prospective cohort studies are needed to confirm its clinical utility and investigate its role in improving treatment strategies and patient management.

## Data Availability

The data analyzed in this study is subject to the following licenses/restrictions: the datasets generated or analyzed during this study are available from the corresponding author upon reasonable request. Requests to access these datasets should be directed to Xinqi Chen, nydxc2010@163.com.
