# Supplementary material for: C-reactive protein to serum calcium ratio as a novel biomarker for predicting severity in acute pancreatitis: a retrospective cross-sectional study
Source: Front Med (Lausanne). 2025 Feb 7;12:1506543. doi: 10.3389/fmed.2025.1506543 (PMC11842247; doi:10.3389/fmed.2025.1506543)
Supplement: Supplementary file 1 [file Table_1.DOC]

Table S1. Univariate logistic regression analysis for moderate to severe acute pancreatitis

| Covariates | Comparisons | OR (95% CI) | P value |
| --- | --- | --- | --- |
| Age |  | 0.97 (0.96~0.99) | <0.001 |
| Gender | Female vs. Male | 0.45 (0.29~0.69) | <0.001 |
| Smoking | Yes vs. No | 1.11 (0.75~1.64) | 0.612 |
| Alcohol consumption | Yes vs. No | 1.43 (0.97~2.1) | 0.068 |
| Diabetes | Yes vs. No | 1.99 (1.31~3.03) | 0.001 |
| Fatty liver | Yes vs. No | 2.8 (1.9~4.11) | <0.001 |
| Etiology | Biliary vs. Hypertriglyceridemia | 0.44 (0.27~0.71) | 0.001 |
|  | Alcohol vs. Hypertriglyceridemia | 0.39 (0.22~0.72) | 0.002 |
|  | Other vs. Hypertriglyceridemia | 0.15 (0.08~0.28) | <0.001 |
| Hospitalization days |  | 1.34 (1.23~1.46) | <0.001 |
| WBC |  | 1 (0.99~1.02) | 0.588 |
| Hemoglobin |  | 1.02 (1.01~1.03) | <0.001 |
| Platelets |  | 1 (1~1) | 0.182 |
| Serum amylase |  | 1 (1~1) | 0.294 |
| Albumin |  | 0.95 (0.91~0.98) | 0.004 |
| AST |  | 1 (1~1) | 0.75 |
| ALT |  | 1 (1~1) | 0.74 |
| Creatinine |  | 1 (1~1.01) | 0.408 |
| Sodium |  | 0.99 (0.97~1.01) | 0.241 |
| Calcium |  | 0.02 (0.01~0.09) | <0.001 |
| CRP |  | 1.03 (1.03~1.04) | <0.001 |
| CCR |  | 1.07 (1.06~1.09) | <0.001 |

WBC, white blood cell; ALT, alanine transaminase; AST, aspartate transaminase; CRP, C-reactive protein; CCR, C-reactive protein/serum calcium ratio; OR, odds ratios; CI, confidence intervals.
